# Identifying roles of “*Jun-Chen-Zuo-Shi*” component herbs of *QiShenYiQi* formula in treating acute myocardial ischemia by network pharmacology

**DOI:** 10.1186/1749-8546-9-24

**Published:** 2014-09-16

**Authors:** Leihong Wu, Yi Wang, Zheng Li, Boli Zhang, Yiyu Cheng, Xiaohui Fan

**Affiliations:** 1 Pharmaceutical Informatics Institute, College of Pharmaceutical Sciences, Zhejiang University, Hangzhou 310058, China; 2 State Key Laboratory of Modern Chinese Medicine, Tianjin University of Traditional Chinese Medicine, Tianjin 300193, China

## Abstract

**Background:**

The role of “*Jun-Chen-Zuo-Shi*” (also known as “sovereign-minister-assistant-courier”) component herbs of Chinese medicine is not fully understood. This study aims to test the “*Jun-Chen-Zuo-Shi*” rule with the *QiShenYiQi* formula (QSYQ) on treating acute myocardial ischemia (AMI) by a network pharmacology approach.

**Methods:**

An Acute Myocardial Ischemia (AMI) specific Organism Disturbed Network (AMI-ODN), was constructed by integrating data of disease-associated genes, protein-protein interaction and microarray experiments. A network-based index, Network Recovery Index for Organism Disturbed Network (NRI-ODN), was developed to measure the therapeutic efficacy of QSYQ and its ingredients, *i.e.*, the ability to recover disturbed AMI network model back to normal state.

**Results:**

The whole formula of QSYQ got a NRI-ODN score of 864.48, which outperformed all individual herbs. Additionally, the primary component herbs, *Radix Astragalus membranaceus* and *Radix Salvia miltiorrrhiza* showed NRI-DON score of 680.27 and 734.31 respectively, which meant a better performance to recover disturbed AMI network than the supplementary component herbs, *Panax notoginseng* and *Dalbergia sissoo* did (545.76 and 584.88, respectively).

**Conclusion:**

AMI-ODN model and NRI-ODN identified the possible roles of “*Jun-Chen-Zuo-Shi*” component herbs of QSYQ in treating AMI at molecular network and pathway level.

## Introduction

Abnormalities in molecular pathways and their interactions have been observed in human diseases [1,2]. Multiple components simultaneously act on multi-targets, such as in the antiretroviral triple cocktail therapy for acquired immunodeficiency syndrome [3], has been considered in drug development. Chinese medicine (CM), featured as ‘multiple ingredients and multiple targets’ [4], is based on “*Jun-Chen-Zuo-Shi*” (also known as “sovereign-minister-assistant-courier”) rule, which explains the complex actions of a CM such as *Indigo naturalis* to treat promyelocytic leukemia [5]. However, the “*Jun-Chen-Zuo-Shi*” rule has not been fully understood and demonstrated in pharmacological science.

Network pharmacology revealed some complicated relationships between diseases and CM [6-10]. In a previous study, we developed network recovery index (NRI) to systemically evaluate drug efficacy by a microarray data annotated biological network [11]. NRI is a quantitative index to measure the ability of a drug to recover perturbed biological network. The NRI measured the synergistic drug efficacy of *SHENMAI* injection consisting of *Red ginseng* and *Radix Ophiopogonis* in treating acute myocardial ischemia (AMI) [11].

*QiShenYiQi* (QSYQ) was a CM prescription for treating ischemic heart disease, while *QI-SHEN-YI-QI* Dropping Pill was approved by the China Food and Drug Administration (CFDA) in 2003 [12]. QSYQ comprises *Radix Astragalus membranaceus* (referred later as Astragalus), *Radix Salvia miltiorrrhiza* (referred later as Salvia), *Panax notoginseng* (referred later as Notoginseng) and *Dalbergia odorifera T. Chen* (referred later as Dalbergia). These herbs constitute the QSYQ formula based upon the principle rule of “*Jun-Chen-Zuo-Shi*”, where Astragalus serves as the sovereign, Salvia serves as the minister, Notoginseng serves as the assistant, and Dalbergia serves as the courier [12,13]. QSYQ exhibited a protective effect comparable to aspirin in preventing myocardial infarction as tested in a double-blind randomized controlled trial [14], QSYQ also significantly reduced infarct size of heart with LAD ligation and increased the density of vessel in ischemic heart in rats [15]. QSYQ could regulate the expressions of proteins associated with energy metabolism in ischemic myocardium of rats [16].

This study aims to test the “*Jun-Chen-Zuo-Shi*” rule of QSYQ in treating AMI by a network pharmacology approach. We developed a network pharmacology based approach, NRI- organism disturbed network (ODN), to investigate the compatibility law of CM formula from both molecule network and pathway level. Firstly, ODN was constructed with disease-related genes, gene expression data and protein-protein interaction (PPI) information to determine the disease systematic influences on the biological network. By combining topology analysis on the ODN and recovery regulation analysis, the gene nodes were weighted by both their topology attributes and recovery ability in regulation of gene expression.

## Methods

### Study workflow

**Figure 1:**
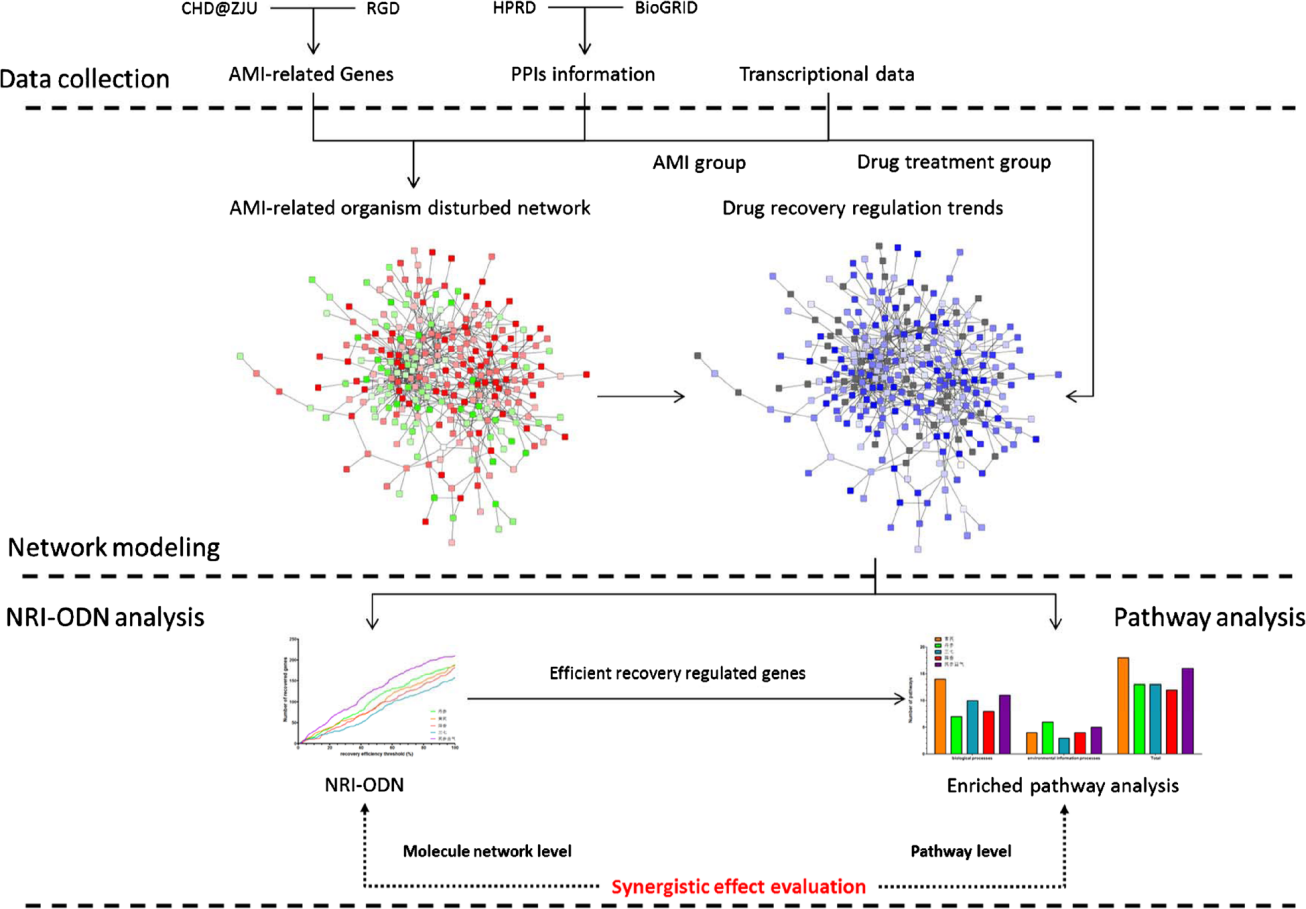
**Study workflow.** Three kinds of information were collected, including AMI-related genes, PPIs information and microarray data to construct the AMI-ODN. The genes in AMI-ODN network were further annotated with recovery regulation of drugs, to visually reveal the recover status of drug on the AMI-ODN. The efficacy of QSYQ and its component herbs were then measured by NRI-ODN, a network based index to evaluating overall recovery ability. On the other side, pathway enrichment analysis was applied on ERGs of QSYQ and its component herbs. As a result, we demonstrated the synergistic effect of QSYQ on treating AMI from molecule network and pathway level.

A study workflow was shown in Figure 1. The AMI-ODN was constructed with AMI-related genes and related PPIs. The network was then annotated with gene expression data. Genes with recovery regulation trend would be evaluated and highlighted (as blue nodes) in the network. NRI-ODN score was used to evaluate the drug efficacy of QSYQ and its component herbs. Additionally, efficient recovery genes (ERGs) from AMI-ODN by QSYQ and its component herbs were calculated, and pathway enrichment analysis with KEGG pathway database was applied to these genes to investigate enriched biological pathways.

### Rat experiment

Male Sprague-Dawley rats (220-250 g) were purchased from Zhejiang Experimental Animal Center (China). The animal experiments were performed according to guidelines of the Animal Ethics Review Committees of Zhejiang University. AMI Rat model was produced through the occlusion of the left anterior descending coronary artery according to Yamaguchi *et al.*[17,18]. The detailed procedure of rat experiment was described by Hong *et al.*[16]. Sham-operated rats with no ligation suture served as control group (Control). Surviving animals were arbitrarily divided into acute myocardial ischemia group (AMI) and QSYQ-treated group, Astragalus -treated group, Salvia-treated group, Notoginseng-treated group and Dalbergia-treated group. Each group included at least 8 rats for calculating a weight ratio of infarct heart tissue calculation and 3 rats for microarray experiments. Instead of using dropping pills, QSYQ extracts, composed by extracts of Astragalus, Salvia, Notoginseng and Dalbergia, was used in this study to investigate the “*Jun-Chen-Zuo-Shi*” rule. The dosage of QSYQ and each component herb extracts used in this work was listed as follows: Astragalus extracts (105.7 mg/kg/day), Salvia extracts (84.9 mg/kg/day), Notoginseng extracts (9.4 mg/kg/day), and volatile oil extracted from Dalbergia (4.7 μL/kg/day). Rats in Control and AMI groups were administered with saline. After treatment for 7 days, rats were sacrificed. The border between infarct and non-infarct left ventricle area in rat heart were harvested to extract mRNA.

### RNA extraction and microarray experiments

Total RNA was extracted and purified by TRIZol Reagent (Invitrogen, USA) and RNeasy Mini kit (QIAGEN, Germany). An Agilent 2100 Bioanalyzer and electrophoresis in 2% (v/v) agarose gels were used for quality assessment of RNA. Only RNA samples with RNA integrity numbers (RINs) over 7.0 and 28SrRNA/18S rRNA greater than 0.7 were used for further microarray experiments. Whole genome microarray analysis was performed using Affymetrix rat Genome 230 2.0 chips (Affymetrix, USA) in accordance with the manufacturer’s protocol. Spike-in control transcripts were monitored to verify hybridization integrity.

### ODN model construction

AMI related gene information is collected from gene knowledgebase such as CHD@ZJU [19] and RGD [20], and the PPI relationships of these genes are collected from public PPI databases HPRD [21] and BioGRID [22], to construct the AMI-ODN. Then, expression value of AMI-related genes is obtained from microarray experiments. Expression value is firstly used as a filter, only genes with considerable intensity and significant expression changes (measured by fold change, equation 1) will be considered in the final AMI-ODN. If a gene had multiple corresponding probes, the largest (in absolute) fold change among all probes was used.

(1) $$\mathrm{FC}={\mathrm{LogE}}_{\mathrm{disease}}-{\mathrm{LogE}}_{\mathrm{control}}$$

where FC indicates the Fold Change, LogE_disease_ indicates the Log_2_ expression value of disease group, and LogE_control_ indicates the Log_2_ expression value of control group.

The details of AMI-ODN construction were as follows. (1) In the step of gene collection, two public databases, CHD@ZJU (Version 1.0; includes 660 coronary heart disease related genes) [19] and Rat Genome Database (RGD; totally 366 genes are reported to be related with myocardial ischemia) [20] cardiovascular portal, were used. By integrating these two database, 820 unique genes are found. (2) In the step of gene filtering, AMI group averaged Log_2_ normalized intensity (aver_Log_2_ > 5) and absolute value of fold change between AMI and control group (abs_FC > 0.2) were used as thresholds to identify considerably and significantly altered expression of AMI-related genes. (3) In the step of PPI information collection, PPIs were collected from two PPI database, HPRD (included 41327 human PPIs with supporting literature evidences) [21] and BioGRID (contained 209838 PPIs) [22]. Only the PPIs that involve AMI-related genes as both source and target genes were used. (4) Finally, the qualified AMI related genes and PPIs were used to construct a AMI-related PPI network by Cytoscape [23], and the AMI-ODN was defined as the biggest sub-network (the giant component).

### NRI-ODN and efficiency of recovery regulation

Network recovery index (NRI) was previously proposed by our group to determine the drug efficacy of CM [11] and was measured according to equation 2a. The recovery regulation and regulation level are measured according to equation 2b and equation 2c. In equation 2c, LogE represents Log2 normalized intensity of experimental group, and equation 2b represents the ratio of genes with ability to recover the AMI network to normal states. NRI indicates the network recovery index, RR indicates recovery regulation, and RL indicates the regulation level.

(2a) $$\mathrm{NRI}={\mathrm{RR}}_{\mathrm{all}}+{\mathrm{RR}}_{\mathrm{up}}+{\mathrm{RR}}_{\mathrm{down}}$$

(2b) $$\mathrm{RR}=\frac{\mathrm{num}\left(\mathrm{RL}>0\right)}{N}$$

(2c) $$\mathrm{RL}=\frac{{\mathrm{LogE}}_{\mathrm{drug}}-{\mathrm{LogE}}_{\mathrm{disease}}}{{\mathrm{LogE}}_{\mathrm{control}}-{\mathrm{LogE}}_{\mathrm{disease}}}$$

The nodes with positive regulation level contribute equally to the final NRI score. The topology and efficiency of recovery regulation are not considered in this study. However, the topology of node described the biological importance of the gene node to the whole ODN, such as the nodes with high degrees in a biological network are probably more important genes in the biological system. On the other hand, the efficiency of recovery regulation (EoR) also could quantitatively indicate the drug ability to recover altered gene expression.

RR was optimized and replaced by RR-ODN to measure the nodes recovery ability by considering their topology and EoR, as equation 3a. The topology weight was the degree centrality of node, which represented the number of directly connected neighboring nodes. The nodes with EoR < 0 (regulation level >2 or <0) would not be used to calculate RR-ODN calculation. Finally the NRI-ODN was measured according to equation 3b.

(3a) $${\mathrm{RR}}_{\mathrm{ODN}}=\sum W_{\mathrm{topo}}*\mathrm{Eo}R_{\mathrm{positive}}$$

(3b) $${\mathrm{NRI}}_{\mathrm{ODN}}={\mathrm{RRODN}}_{\mathrm{all}}+{\mathrm{RRODN}}_{\mathrm{up}}+{\mathrm{RRODN}}_{\mathrm{down}}$$

where W indicates the topology weight parameter, RR_ODN_ indicates the new recovery regulation index in NRI-ODN.

EoR was the efficiency of a drug to recover disturbed gene as defined in equation 4. 50% EoR would mean that the drug could recover the gene dysregulation by half (7.5) or 50% over-regulated the gene expression (12.5); 100% EoR would mean that the drug could fully recover the gene expression from disease to control state (10). In this study, EoR ≥ 50% was used as a threshold of identify efficient recovery regulated genes (ERGs).

(4) $$\mathrm{EoR}=100\%-\left|100\%-\mathrm{RL}\right|$$

### Pathway enrichment analysis

Pathway enrichment analysis with KEGG pathway database [24] was applied on ERGs of QSYQ and its component herbs, in which the ERGs was defined as the genes with EoR > 50%. By using ArrayTrack [24], an enriched pathway represented those pathways which had a Fisher *P* value <0.05. Only the pathways of cellular processes and environmental information processes were considered.

## Results

### The weight ratio of infarcted heart tissue

**Figure 2:**
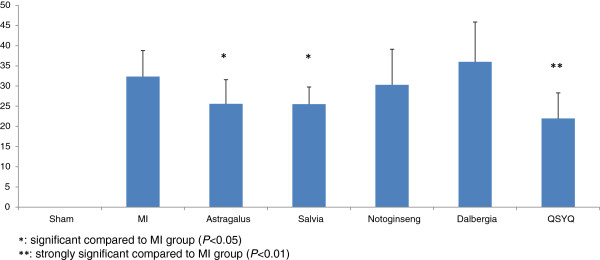
**Statistics of the weight ratio of infarcted heart tissue in rat experiments.** QSYQ reduced the weight ratio of infarcted heart tissue more significantly than its component herbs, *i.e.*, Astragalus, Salvia, Notoginseng and Dalbergia, did.

Therapeutic effects of QSYQ treatment and each individual component treatment were evaluated by the weight ratio of infarcted heart tissue. As shown in Figure 2, the AMI injury was quantitatively evaluated for each group by the weight ratios between infarcted zone and the whole tissue. The QSYQ showed efficacy on treating AMI by significantly reducing the weight ratio of infarcted heart tissue (*P* = 0.002), with a lower *P* value than any of its component. Among four component herbs, Astragalus and Salvia also showed significant effect on treating AMI (*P* = 0.02 and 0.01, respectively), but Notoginseng and Dalbergia showed insignificant effects (*P* > 0.1).

### AMI-ODN construction

**Figure 3:**
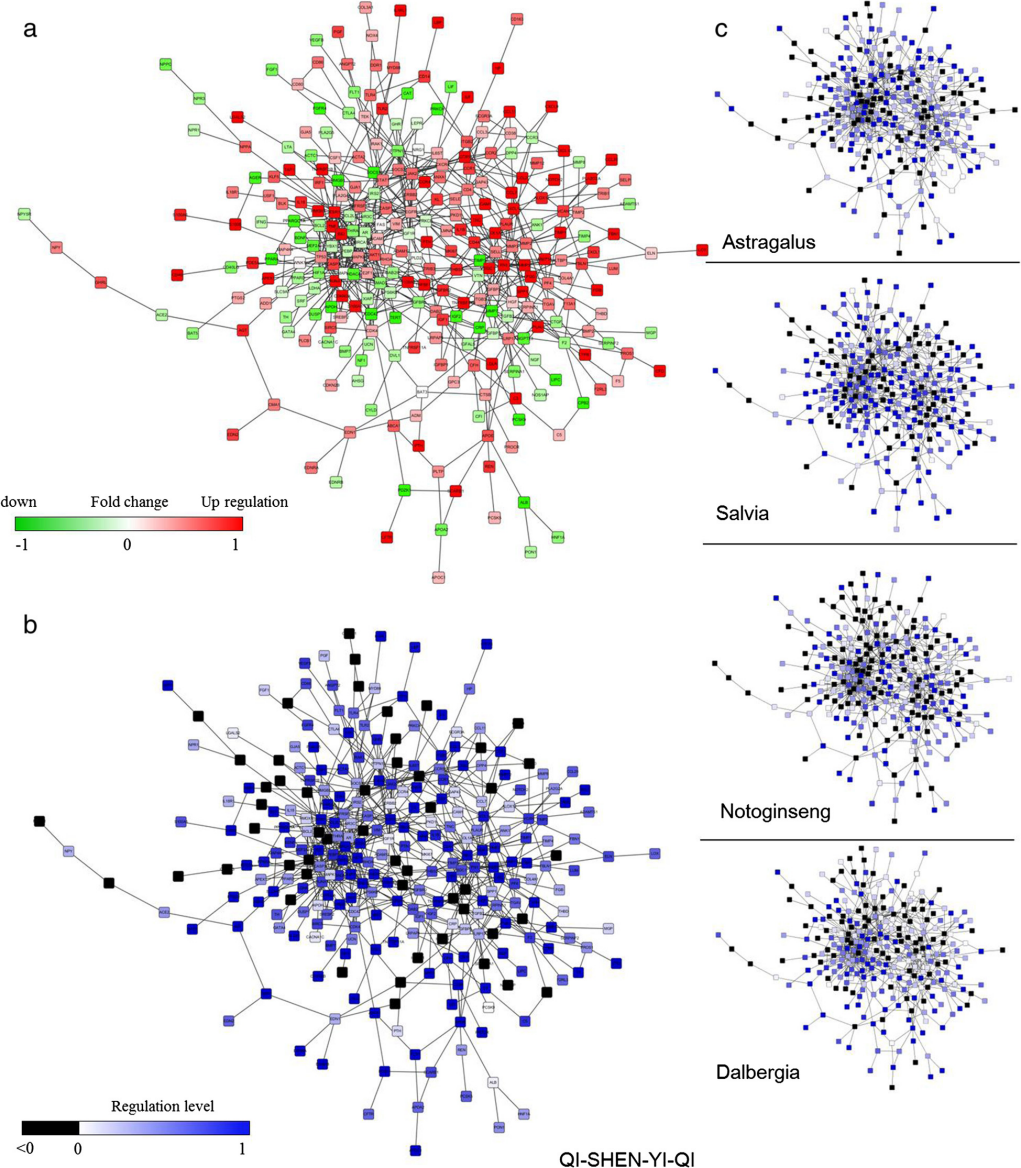
**Recovery regulation of QSYQ and its components on AMI-ODN. (a)** The AMI-ODN contained 281 nodes and 616 edges. Node was annotated with its expression fold changes between AMI and control group, red indicated up-regulation and green indicated down-regulation. **(b)** Recovery regulation of QSYQ on AMI-ODN. Nodes in AMI-ODN with recovery regulation were colored in blue, indicating that QSYQ could recover this gene node from disease to control state. Grey nodes represented a negative regulation level. **(c)** Recovery regulation of Astragalus, Salvia, Notoginseng and Dalbergia on AMI-ODN.

By integrating gene information from CHD@ZJU and RGD, 820 AMI related genes were found. Fold change >0.2 and average Log2 normalized intensity >5 were then used to find significant expressed and changed genes in the microarray experiments, and PPIs among these genes were found from HPRD and BioGRID databases. As a result, 623 PPI interactions among 324 AMI related genes were used to construct the AMI-related PPI network. The biggest sub-network contained 281 genes and 616 PPI relationships, which were then defined as the AMI-ODN (Figure 3a).

We calculated the regulation level of QSYQ on the AMI-ODN, and annotated the node color to show the recovery status (Figure 3b). The genes with positive regulation, indicating recovered regulation, were colored in blue. The regulation levels of component herbs were also visualized as shown in Figure 3c. Apparently, QSYQ could affect most of the nodes in the ODN which was disturbed in the disease state.

### Evaluating efficacy and synergistic effect of QSYQ

The NRI-ODN was then measured to quantitatively evaluate the drug efficacy of QSYQ and its component herbs (Table 1). For instance, the RR-ODN of QSYQ for all network, significantly down-regulated nodes and significantly up-regulated nodes were 513.03, 78.45 and 273.00, respectively. The score of NRI-ODN of QSYQ was 864.48, which showed better recovery performance compare to its components. Among the four component herbs, Salvia showed the highest NRI-ODN (734.31) which was slightly higher than that of Astragalus (680.27), whereas Notoginseng (545.76) and Dalbergia (584.88) showed relatively lower NRI-ODN scores. According to the “*Jun-Chen-Zuo-Shi*” rule, Astragalus (Sovereign) and Salvia (Minister) were expected to recover disturbed network better than Notoginseng (Assistant) and Dalbergia (Courier) did, which was accordance to the NRI-ODN result.

**Table 1:** NRI-ODN results of QSYQ and its component herbs

	**QSYQ**	**Astragalus**	**Salvia**	**Notoginseng**	**Dalbergia**
RR-ODN (All)	513.03	399.40	432.18	318.14	362.31
RR-ODN (Down)	78.45	74.59	80.96	48.50	76.77
RR-ODN (Up)	273.00	206.28	221.17	179.12	145.81
NRI-ODN	864.48	680.27	734.31	545.76	584.88
*P* value	1.84E-12**	1.82E-04**	2.45E-06**	0.185	0.042*

**Extremely significant (<0.01).

*significant (<0.05).

A permutation test was performed to check whether QSYQ and its component herbs recovered ODN network more significantly than any random permutation in ODN with a normal distribution. The NRI-ODN of each permutation was calculated and repeated 10000 times. As a result, the average value of permutated NRI-ODN was 471.96, and the standard deviation was 55.64 in 10000 repetitions. The *P* value of NRI-ODN of QSYQ and its component herbs was calculated from the permutation test (Table 1). Astragalus and Salvia showed a highly significant recovery from a disturbed network (*P* < 0.01), and Dalbergia showed a significant recovery (*P* < 0.05), while the recovery of Notoginseng on ODN was not statistically significant (*P* = 0.18).

### The effect of fold change threshold on NRI-ODN calculation

**Figure 4:**
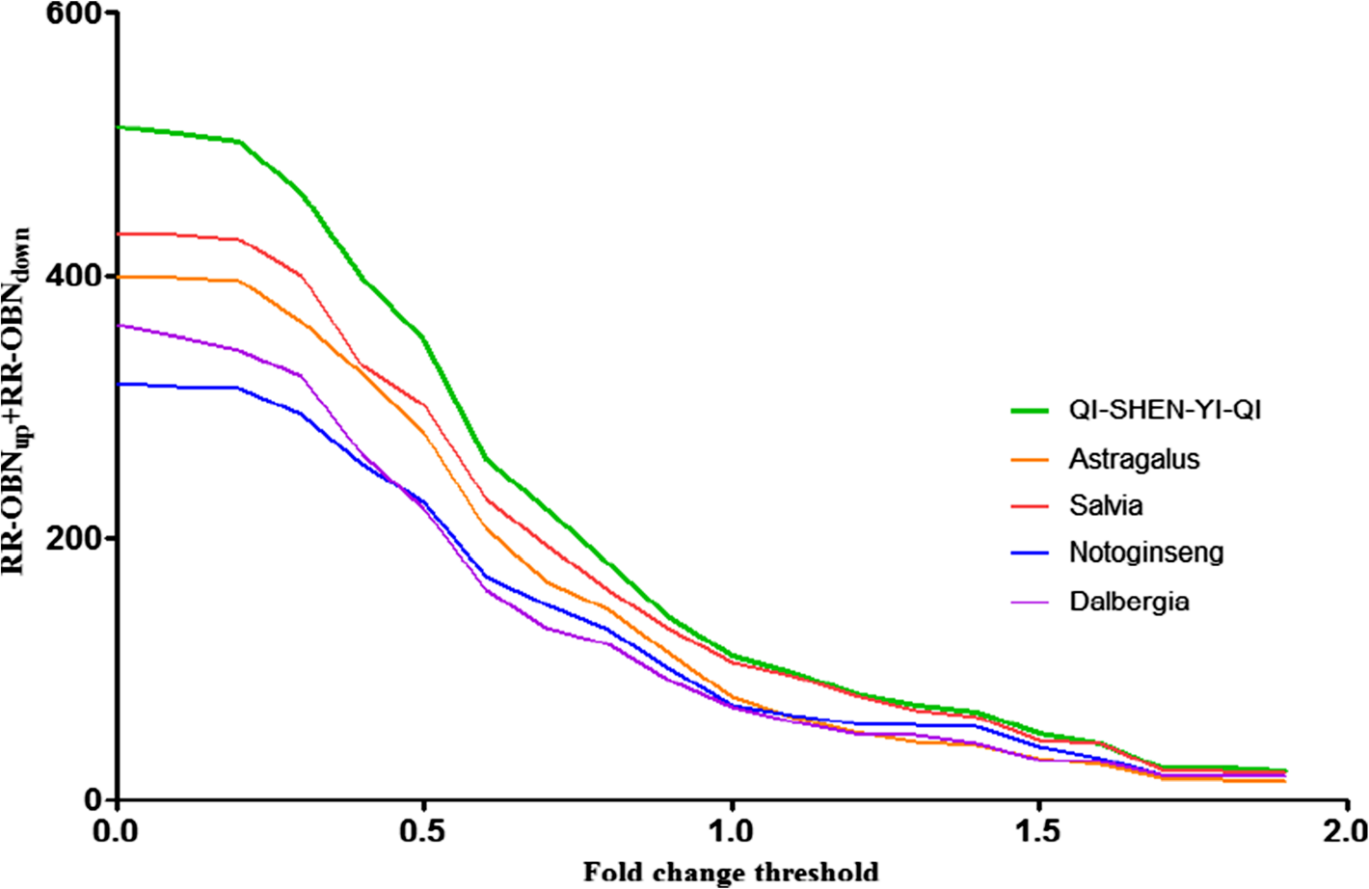
**The RR-ODN with top N genes (ranged from 1 to 281) sorted by absolute fold changes.** The x-axis represents number of genes and y-axis represents RR-ODN of QSYQ and its component herbs. QSYQ showed the better RR-ODN than its individual component herbs. Among four component herbs, Astragalus and Salvia showed higher RR-ODN than Notoginseng and Dalbergia, in accordance with their primary roles in QSYQ.

In NRI-ODN calculation, the up-regulated and down-regulated genes were identified by fold changes. The effect of fold change thresholds on the RR-ODN calculation was investigated. In Figure 4, the x-axis represented the threshold of fold change. For instance, x = 0.1 is the threshold to identify the up-regulated genes with fold change >0.1 and the down-regulated genes with fold changes < -0.1. In particular, x = 0 meant the same as RR-ODN_all_. The y-axis was the total significantly regulated RR-ODN score which contributed by both up-regulated and down-regulated genes. The green line represented the RR-ODN of QSYQ, and the orange, red, blue and purple lines represented the RR-ODN of Astragalus, Salvia, Notoginseng and Dalbergia, respectively. The overall RR-ODN of QSYQ and its component herbs were consistent when fold change threshold <1. The performance of QSYQ was more effective than any individual component herb. The performances of Astragalus and Salvia were equivalent and significantly better than performances of Notoginseng and Dalbergia. When fold change threshold was set >1, there were fewer genes (only 34 genes remained with fold change >1) which RR-ODN result was seriously influenced by any single node’s performance. Therefore, fold change =0.5 was used as the threshold to identify significantly regulated genes. In this study, 169 significantly regulated genes were found.

### Pathway enrichment analysis

ERGs were defined by EoR > 50%. There were 131, 90, 111, 76 and 89 ERGs in the ODN for QSYQ, Astragalus, Salvia, Notoginseng and Dalbergia, respectively. Pathway enrichment analysis was conducted on the effective regulation recovery genes of QSYQ and its component herbs. The number of significant enriched pathway (Fisher’s p-value <0.05) of QSYQ was significantly more than its component herbs, Salvia, Notoginseng and Dalbergia (Table 2). However, Astragalus had more enriched pathways than QSYQ did.

**Table 2:** Number of enriched pathways of QSYQ and its component herbs

	**QSYQ**	**Astragalus**	**Salvia**	**Notoginseng**	**Dalbergia**
Number of ERGs	131	90	111	76	89
Cellular processes	11	14	7	10	8
Environmental information processes	5	4	6	3	4
Total biological pathways	16	18	13	13	12
Overlapped pathways	--	10	10	8	5

The numbers of overlapping pathways between QSYQ and a component herb including Astragalus, Salvia, Notoginseng, and Dalbergia were 10, 10, 8 and 5, respectively. The result indicated that Salvia and Notoginseng acted on p53 pathways whereas Astragalus and Dalbergia did not. It suggested that the effect of QSYQ on p53 signaling pathway was attributed to Salvia and Notoginseng. Detailed pathway information of QSYQ and its component herbs were provided in Table 3.

**Table 3:** Enriched pathway results of QSYQ and its component herbs

**Pathway Name**	**KEGG ID**	**QSYQ**	**Salvia**	**Astragalus**	**Notoginseng**	**Dalbergia**
Complement and coagulation cascades	hsa04610	1	1	1	1	1
Cytokine-cytokine receptor interaction	hsa04060	1	1	1	1	1
MAPK signaling pathway	hsa04010	1	1	1	1	1
Chemokine signaling pathway	hsa04062	1	1	1	1	0
Focal adhesion	hsa04510	1	1	1	0	1
Adipocytokine signaling pathway	hsa04920	1	1	1	0	0
Endocytosis	hsa04144	1	1	0	1	1
TGF-beta signaling pathway	hsa04350	1	1	0	1	0
p53 signaling pathway	hsa04115	1	1	0	1	0
ECM-receptor interaction	hsa04512	1	1	0	0	0
Vascular smooth muscle contraction	hsa04270	1	0	1	1	0
Apoptosis	hsa04210	1	0	1	0	0
PPAR signaling pathway	hsa03320	1	0	1	0	0
Toll-like receptor signaling pathway	hsa04620	1	0	1	0	0
Cell adhesion molecules	hsa04514	1	0	0	0	0
Regulation of actin cytoskeleton	hsa04810	1	0	0	0	0

Out of 16 pathways, three of them, as complement and coagulation cascades, cytokine-cytokine receptor interaction and MAPK signaling pathway, were enriched in QSYQ and all its four component herbs. On the other hand, two pathways, *i.e.*, cell adhesion molecules and regulation of actin cytoskeleton, were specially enriched by QSYQ, which might imply novel function of component combination.

As shown in Table 3, all enriched pathways of Notoginseng and Dalbergia were at least enriched in either Astragalus or Salvia, which suggested that Notoginseng and Dalbergia might play roles as *Zuo* (assistants) that would not bring new pathway regulation into QSYQ, but to enforce regulating effects of Astragalus and Salvia enriched pathways.

In Table 3, vascular smooth muscle contraction, apoptosis, PPAR signaling pathway and Toll-like receptor signaling pathway are enriched by Astragalus but not Salvia. Among them, vascular smooth muscle contraction reflected the roles of vascular smooth muscle cell (VSMC) to regulate the blood flow and pressure [25]. PPAR signaling pathway was highly correlated with lipids metabolism and adipocyte differentiation. These two enriched pathways might indicate the Astragalus would play roles on energy metabolism to relieve the blood flow dysregulation caused by acute myocardial ischemia. Apoptosis and Toll-like receptor signaling pathway were correlated with cell death and immune system [26,27], which implied potential roles of Astragalus on these biological functions. Astragalus regulated BCL-2 protein family (such as BCL-2 and BAX), which was critical in apoptosis and correlated with p53 and TGF-beta signaling pathway [28]. Astragalus also reduced expression of BAX in heart failure rats and increase Bcl-2 expression by Astragaloside IV, a main component in Astragalus [29,30].

On the other side, endocytosis, TGF-beta signaling pathway, p53 signaling pathway and ECM-receptor interaction were enriched by Salvia but not Astragalus. Activation of TGF-beta signaling pathway was found in the infarcted myocardium with potential effects on repression of inflammatory genes and mediating resolution of the inflammatory infiltrate TGF-beta also mediates events in the pathogenesis of hypertrophic and dilative ventricular remodeling [31]. P53 signaling pathway was induced by stress signals, such as DNA damage, oxidative stress, and was correlated with apoptosis and cell cycle arrest [32]. The enrichment of TGF-beta signaling pathway and p53 signaling pathway indicated the Salvia might play important roles in apoptosis, which was also regulated by Astragalus.

Furthermore, Astragalus, Salvia and Notoginseng could regulate Chemokine signaling pathway together, which was highly correlated with inflammation processes [33]. In all, the QSYQ might gain enhanced drug effect by regulating apoptosis and inflammation related pathways together.

## Discussions

In this study, we integrated AMI-related gene and its expression data, with PPI information to construct AMI-ODN model, and further applied the network model to reveal the compatibility law of QSYQ on treating AMI. In particular, the network recover ability of QSYQ and its component herbs were quantitatively measured by NRI-ODN to demonstrate that the synergistic efficacy of QSYQ on treating AMI. Among four component herbs, Astragalus and Salvia showed stronger recovery ability than Notoginseng and Dalbergia, which was also conformed to the “*Jun-Chen-Zuo-Shi*” rule of QSYQ.

The primary roles of Astragalus and Salvia in QSYQ were further validated at pathway level. By pathway enrichment analysis, Astragalus and Salvia could regulate more pathways and play special roles on unique pathways, such as Astragalus could regulate energy metabolism to relieve the blood flow caused by myocardial ischemia. The combination of Astragalus and Salvia could also enhance the regulating ability of important biological processes, such as apoptosis and inflammation system.

The successful application of network-based approaches on QSYQ was more sensitive than traditional, phenotype-based evaluating method, such as the weight of infarcted zones. More importantly, the synergistic effect and contribution of drug efficacy could now be evaluated from a molecule network and pathway level, which was further related to its underlying “multi-target, multi-processes” mechanisms.

ODN modeling and evaluation with NRI-ODN score were suitable for studying high-throughput data and complex diseases which involved multiple genes. It might have limited performance for orphan diseases. ODN modeling was independent of chemical structures, and suitable for discovering the similar and distinct effects between CM and chemical drugs.

## Conclusion

AMI-ODN model and NRI-ODN identified the possible roles of “*Jun-Chen-Zuo-Shi*” component herbs of QSYQ in treating AMI at molecular network and pathway level.

## Abbreviations

AMI: Acute myocardial ischemia; AMI-ODN: Acute myocardial ischemia organism disturbed network; CM: Chinese medicine; EoR: Efficiency of recovery regulation; ERG: Efficient recovery regulated genes; NRI: Network recovery index; NRI-ODN: Network recovery index for organism disturbed network; ODN: Organism disturbed network; QSYQ: *QiShenYiQi* formula; RL: Regulation level; RR: Recovery regulation; RR-ODN: Recovery regulation for organism disturbed network.

## Competing interests

The authors declare that they have no competing interests.

## Authors’ contributions

XF, ZL, BZ and YC conceived and designed the study. YW performed the rat experiments. LW and XF analyzed the transcriptional data and performed network analysis. LW, XF and ZL wrote the manuscript. All the authors read and approved the final manuscript.
